# Retrospective Radiology Research: Do We Need Informed Patient Consent?

**DOI:** 10.1007/s11673-024-10368-6

**Published:** 2024-08-19

**Authors:** Yfke Ongena, Thomas C. Kwee, Derya Yakar, Marieke Haan

**Affiliations:** 1 Centre for Language and Cognition, Discourse and Communication Group, University of Groningen, Oude Kijk in ’t Jatstraat 26, 9712 EK Groningen, The Netherlands; 2 Department of Radiology, Medical Imaging Center, University Medical Center Groningen, University of Groningen, Groningen, The Netherlands; 3 Department of Sociology, University of Groningen, Groningen, The Netherlands

**Keywords:** Ethics, Informed consent, Radiology, Retrospective studies

## Abstract

While knowledge of the population’s view on the need for informed consent for retrospective radiology research may provide valuable insight into how an optimal balance can be achieved between patient rights versus an expedited advancement of radiology science, this is a topic that has been ignored in the literature so far. To investigate the view of the general population, survey data were collected from 2407 people representative of the Dutch population. The results indicate that for non-commercial institutions, especially hospitals (97.4 per cent), respondents agree with the retrospective use of imaging data, although they generally indicate that their explicit consent is required. However, most respondents (63.5 per cent) would never allow commercial firms to retrospectively use their imaging data. When including only respondents who completed the minimally required reading time of 12.3 s to understand the description about retrospective radiology research given in the survey (n = 770), almost all (98.9 per cent) mentioned to have no objections for their imaging data to be used by hospitals for retrospective research, with 57.9 per cent indicating their consent to be required and 41.0 per cent indicating that explicit patient consent to be unnecessary. We conclude that the general population permits retrospective radiology research by hospitals, and a substantial proportion indicates explicit patient consent to be unnecessary when understanding what retrospective radiology research entails. However, the general population’s support for the unrestricted retrospective use of imaging data for research purposes without patient consent decreases for universities not linked to hospitals, other non-commercial institutions, government agencies, and particularly commercial firms.

**Supplementary Information:**

The online version contains supplementary material available at 10.1007/s11673-024-10368-6.

## Introduction

Research is crucial to advance healthcare, including radiology. Prospective studies, in which subjects undergo a medical imaging procedure that is not part of routine clinical care, require ethical review board approval and informed patient consent (Junod and Elger 2010; Stefánsson, Atladóttir and Gudbjornsson 2008). For retrospective research with previously acquired medical imaging data, ethical review board approval is also considered necessary (Junod and Elger 2010; Stefánsson et al. 2008; De Lange et al. 2019). However, there is no consensus about the need for informed patient consent for retrospective research, with widely varying practices between different countries and institutions (Junod and Elger 2010; Stefánsson et al. 2008; De Lange et al. 2019).

Proponents of informed patient consent for retrospective research argue that this is required to respect patient autonomy and privacy regulations (Junod and Elger 2010; Stefánsson et al. 2008; De Lange et al. 2019; “The HIPAA Privacy Rule” 2022; European Commission 2022). Opponents argue that refraining from informed patient consent will decrease unnecessary bureaucracy and expedite research and knowledge acquisition that may benefit patient care (Junod and Elger 2010; Stefánsson et al. 2008; De Lange et al. 2019). Approaching patients for informed consent could also lead to undesirable situations when a patient suffers from a serious disease or has already deceased. Furthermore, it can be argued that medical imaging data can be adequately anonymized or pseudonymized to respect a patient’s privacy (Milchenko and Marcus 2013). Finally, it can be considered practically impossible to contact many patients in large-scale studies (Junod and Elger 2010; Stefánsson et al. 2008; De Lange et al. 2019). The latter is an important issue in the development of artificial intelligence algorithms that need to be trained with large amounts of data (Pinto Dos Santos and Baeßler, 2018).

So far, the view of the general population on the need for informed consent for retrospective radiology research has not been explored. Gaining knowledge of the population’s view may provide valuable insight into how an optimal balance can be achieved between patient rights versus an expedited advancement of radiology science through retrospective research. It is hypothesized that the majority of subjects have no objections when their deidentified medical imaging data are reused for retrospective research purposes.

The purpose of this study was therefore to investigate the view of the general population on the need for informed patient consent for retrospective radiology research.

## Methods

### Study Design and Subjects

For this study we collected data in the September 2022 wave of the online LISS Panel (Longitudinal Internet Studies for the Social Sciences). The LISS panel is a nationally representative household panel study for people aged sixteen years and above in the Netherlands. In a subsample (see Table 1 for demographics) we only included panel members of eighteen years and above, and only one member per household for households that have multiple participating members. The informed consent procedure in the LISS panel ensures double consent, via a reply card and an internet login (see Scherpenzeel and Das 2010 for more details). Ethical approval for the procedures in the LISS Panel was given by the board of overseers (LISS Board of Overseers 2022). For the September 2022 wave, 3425 panel members were contacted and 2407 completed the full questionnaire resulting in a 70.3 per cent response rate. Respondents could fill out the survey on a PC, tablet, or smartphone.

**Table 1:** Demographic characteristics of the sample

	n (%)		Mean (sd)
Age			50.9 (17.5)
Gender			
Male	1157 (48.1%)		
Female	1246 (51.9%)		
Level of education			
Primary	428 (17.8%)		
Secondary	823 (34.2%)		
Tertiary	1093 (45.4%)		
Other	64 (2.7%)		
Experience with…	diagnostic radiology	interventional radiology	a family member undergoing radiological diagnostic test or treatment
Yes	1778 (73.9%)	217 (9.0%)	612 (25.4%)
No	527 (21.9%)	2117 (88.0%)	1702 (70.7%)
I don’t know	67 (2.8%)	41 (1.7%)	57 (2.4%)
I’d rather not say	35 (1.5%)	32 (1.3%)	36 (1.5%)
Last time experience with…	diagnostic radiology	interventional radiology	a family member undergoing radiological diagnostic test or treatment
This year	345 (19.4%)	33 (15.2%)	119 (19.4%)
Last year	217 (12.2%)	26 (12.0%)	87 (14.2%)
Longer than a year ago	1137 (63.9%)	150 (69.1%)	387 (63.2%)
I don’t know	77 (4.3%)	7 (3.2%)	16 (2.6%)
I’d rather not say	2 (0.1%)	1 (0.5%)	3 (0.5%0

### The Questionnaire

To investigate the general population’s view on the need for informed patient consent for retrospective radiology research, we included eight questions in the September 2022 wave asking respondents about the re-use of imaging data for this purpose and their experience with clinical radiology. Prior to these questions, respondents were asked several other questions about diagnostic and interventional radiology that were not part of the present study.

### Measurement of Agreement with Retrospective Use of Imaging Data for Research

Respondents were first given an explanation of retrospective radiology research and the use of anonymized imaging data for research. Time spent on reading the explanation was measured with time stamps. The text for this explanation was:

Next, we will ask a number of general questions about conducting radiological research with anonymized data (for example, research with X-rays and images from a CT scan). Anonymized means that the data do not contain recognizable personal data of patients. The researchers can therefore not identify the people to whom the data belong.

Hundreds of radiological examinations are performed in every hospital every day. The corresponding images are stored in every hospital, and can be reused for research.

The original Dutch version of the text included eighty words, with an average word length of six letters. Given the fact that the average reading speed of Dutch texts with an average word length of six letters is 202 words per minute (Brysbaert, et al. 2021), we assumed that it would take an average of twenty-four seconds to read the explanation, and the minimum required time for fast readers would be 12.3 s (two standard errors below the average reading time). A separate analysis was conducted for respondents who spent at least 12.3 s on reading the explanation. After the explanation respondents were given the question “If a patient has undergone a diagnostic imaging study, by whom do you think the anonymized information/data from this study may be reused?” Respondents could indicate per type of institution (hospital, university, other non-commercial institution, commercial firms, governmental agencies) whether this institution could use the data; (option 1) without approval of the patient (“permission without explicit patient consent”), (option 2) only with approval of the patient (“permission with explicit patient consent”), or (option 3) not at all (“never permission”). The different types of institutions were offered in a randomized order to counterbalance order effects. The same question was also asked for the interventional radiology setting: “If a patient has undergone an interventional radiology procedure, by whom do you think the anonymized information/data from this study can be reused?”.

### Measurement Predictor Variables

As potential predictor variables we investigated gender, level of education, and experience with radiological procedures (either as a patient or as a person accompanying a patient). Level of education was measured with the LISS-panel item of highest earned degree, and for purposes of analysis, categories taken from the Dutch educational system were converted into international categories; primary education, secondary (i.e., pre-university education or mediate vocational education), tertiary (i.e., university or higher vocational education) and other (no degree or degree not included among response options). Respondents were asked whether they had experience with diagnostic radiology, with interventional radiology, and/or being a representative of a friend, acquaintance or family member undergoing imaging studies (for diagnosis or treatment) at a radiology department. If respondents had indicated experience with any of these three situations, they were subsequently asked whether their last experience occurred in the current year, last year, or longer ago.

## Statistical Analysis

Agreement of retrospective use of imaging data was measured with categorical response options that are incremental in terms of agreement (i.e., allowing retrospective use of imaging data *without* explicit approval of the patient is less restrictive than allowing use only *with* explicit approval, and both are less restrictive than *never* allowing retrospective use of imaging data). Because of the ordinal level of measurement, we cannot assess the exact level of agreement numerically. Therefore, an ordinal logistic regression was used to assess the effects of the predictor variables on the three levels of agreement of retrospective use of imaging data. To interpret odds ratios we used: > 1.5 (small effect), > 2 (medium effect), > 3 (large effect) and for negative effects the multiplicative inverse (1/x) of the classification was used: < 0.66 (small), < 0.5 (medium), < 0.33 (large) (Sullivan and Feinn, 2012).

## Results

### Descriptive Statistics

The responses to the question “If a patient has undergone a diagnostic imaging study, by whom do you think the anonymized information/data from this study can be reused?” showed that for non-commercial institutions, especially hospitals (97.4 per cent), respondents agree with the retrospective use of imaging data, although they generally indicate that their explicit consent is required. However, the majority of respondents (63.5 per cent) would never allow commercial firms to retrospectively use their imaging data (Table 2). Responses were analysed separately for respondents with a reading time of 12.3 s or more, to ensure that the description about retrospective radiology research was sufficiently read to be understood. After removing outliers above 1.5 times the interquartile range, the average reading time was 10.1 s (sd = 9.9), and 75 per cent of respondents had a reading time of 16 s or lower. Reading times were significantly different for age (for each year, the amount of time increased with 0.18 s) and education (respondents with a tertiary level of education spent 1.34 times more time on reading than respondents with primary education). When including only respondents (n = 770) with the minimally required reading time of 12.3 s or more, almost all (98.9 per cent) mentioned to have no objections for their imaging data to be used by hospitals for retrospective research, with 57.9 per cent indicating their consent to be required and 41.0 per cent indicating that explicit patient consent to be unnecessary. Note that the size of the latter group (i.e., approval without explicit patient consent) increased by 10 percent points relative to the entire group of respondents (including those with a reading time of less than 12.3 s), and similar increases were found for the other institutions. Responses to the question “If a patient has undergone an interventional radiology procedure, by whom do you think the anonymized information/data from this study can be reused?” showed exactly the same pattern (Supplemental materials, Table 2a).

**Table 2:** Descriptive statistics of consent with retrospective use of diagnostic imaging data

	All respondents (n = 2409)			Respondents with reading time > 12.3 s (n = 770)		
	Never permission	Permission *with* explicit patient consent	Permission *without* explicit patient consent	Never permission	Permission *with* explicit patient consent	Permission *without* explicit patient consent
By the hospital	62 (2.6%)	1589 (66.0%)	757 (31.4%)	8 (1.0%)	446 (57.9%)	316 (41.0%)
By a university	248 (10.3%)	1764 (73.3%)	396 (16.4%)	51 (6.6%)	524 (68.1%)	195 (25.3%)
By other non-commercial institutions	423 (17.6%)	1653 (68.6%)	332 (13.8%)	99 (12.9%)	497 (64.5%)	174 (22.6%)
By commercial firms	1529 (63.5%)	823 (34.2%)	56 (2.3%)	503 (66.0%)	238 (30.9%)	24 (3.1%)
By government agencies	493 (20.5%)	1525 (63.3%)	390 (16.2%)	144 (18.7%)	463 (60.1%)	163 (21.2%)

### Ordinal Logistic Regression

Table 3 presents the results of the ordinal logistic regression analysis that was conducted to examine the relationship between various predictor variables and the outcome variable of consent with retrospective use of data in five types of institutions. According to the Akaike information criterion (AIC) and residual variance this model had significant better fit to the data as compared to the same analysis without interaction effects (LR(df = 3) = 26.32, p < 0.001). The table presents the ORs for each predictor variable, along with the corresponding confidence intervals (CIs) and p-values. The analysis shows that “never permission” was far less likely to be chosen than “permission with explicit patient consent,” whereas “permission without explicit patient consent” was much more likely to be chosen than “permission without explicit patient consent.” Furthermore, the institutions (taking the hospital as reference category) have medium negative effects (university; OR = 0.41) or large negative effects (commercial, non-commercial, and government, OR = 0.27, 0.03, 0.26, respectively); i.e., consent is higher for use of data by hospitals than by other types of institutions. Effects of education showed that respondents with secondary and tertiary education were more likely to choose “without approval” than respondents with primary education, showing a medium effect size (OR = 2.41 for tertiary education) and a small effect size (OR = 1.61 for secondary education). In addition, interaction effects between gender and education showed small effects for females with secondary education (OR = 0.56) and tertiary education (OR = 0.62). The odds of choosing “permission without explicit patient consent” were lower for females with secondary or tertiary education, compared to females with primary education, while these odds were higher for males with secondary or tertiary education than for males with primary education. A significant positive effect was found for reading time (the amount of time respondents had spent on reading the explanation of retrospective radiology and storage of anonymized imaging data). Significant negative effects (though negligible in effect size) were found for experience with radiological examinations (respondents with no experience were less likely to choose “permission without explicit patient consent than respondents with experience) and age (the odds of choosing “permission without explicit patient consent” slightly decreased with increasing age). Separate ordinal logistic regression analyses per institution (Table 4) showed that the effect of reading time did not hold for responses for the item on commercial institutions, the effect of education (tertiary versus primary) was largest for the item on the government (OR = 3.92), and the effect of no experience was largest for the item on hospitals, though still a small effect (OR = 0.66). The interaction effect of gender and education was strongest (though still small) for the items on non-commercial institutions, commercial organizations, and the government.

**Table 3:** Ordinal logistic regression analysis on approval and need for consent for retrospective radiology research in five types of institutions, institutions as fixed effects

	Odds Ratios	CI	p
Never Permission|With explicit consent	0.21	0.07—0.60	**0.004**
With explicit consent|Without explicit consent	8.59	2.96—24.96	** < 0.001**
University	0.41	0.36—0.46	** < 0.001**
Non-commercial	0.27	0.24—0.31	** < 0.001**
Commercial	0.03	0.02—0.03	** < 0.001**
Government	0.26	0.23—0.30	** < 0.001**
Gender (female)	1.03	0.84—1.26	0.777
Reading time	1.03	1.02—1.03	** < 0.001**
Education (ref = Primary)			
Secondary	1.61	1.34—1.93	** < 0.001**
Tertiary	2.41	2.03—2.87	** < 0.001**
Other education	1.19	0.81—1.74	0.381
Experience with radiological exam (ref = before 2021)			
Experience in 2021	0.89	0.77—1.03	0.123
Experience in 2022	1.00	0.89—1.13	0.973
No experience	0.82	0.74—0.91	** < 0.001**
Age	0.99	0.99—0.99	< 0.001
Female* secondary educ	0.56	0.44—0.72	< 0.001
Female* tertiary	0.62	0.49—0.78	< 0.001
Female*other educ	1.14	0.65—2.00	0.642
Residual variance	2847.41		
AIC	2875.41

**Table 4:** Odds ratios from ordinal logistic regression analyses on approval and need for consent for retrospective radiology research per institution

	Hospital	University	Non-commercial	Commercial	Government
Gender (female)	0.78	1.02	1.08	1.06	1.19
Reading time	1.04***	1.04***	1.04***	1.00	1.02***
Education (ref = Primary)					
Secondary	1.50*	1.26	1.81**	1.45	2.01**
Tertiary	1.92**	2.48***	2.27***	1.89**	3.92***
Other education	0.70	1.47	1.37	1.52	0.98
Experience with radiological exam (ref = before 2021)					
Experience in 2021	0.94	1.05	0.83	0.96	0.75
Experience in 2022	0.94	1.02	1.07	1.14	0.88
No experience	0.66***	0.71**	0.84	1.10	0.79*
Age	0.99***	0.99*	1.00	0.98***	0.99***
Female* secondary educ	0.66	0.68	0.52 *	0.52*	0.50**
Female* tertiary	0.83	0.64	0.61	0.57*	0.51**
Female*other educ	2.48	0.34	1.55	0.64	2.14

*p < 0.05 (2-tailed), ** p < 0.01 (2-tailed), *** p < 0.001 (2-tailed)

**Fig. 1:**
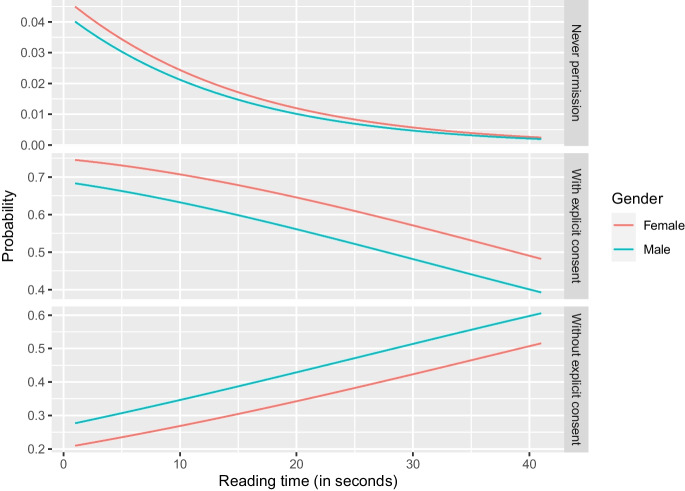
Should patients approve use of retrospective data by a hospital? Specified for gender and reading time of an explanation of retrospective radiology

**Fig. 2:**
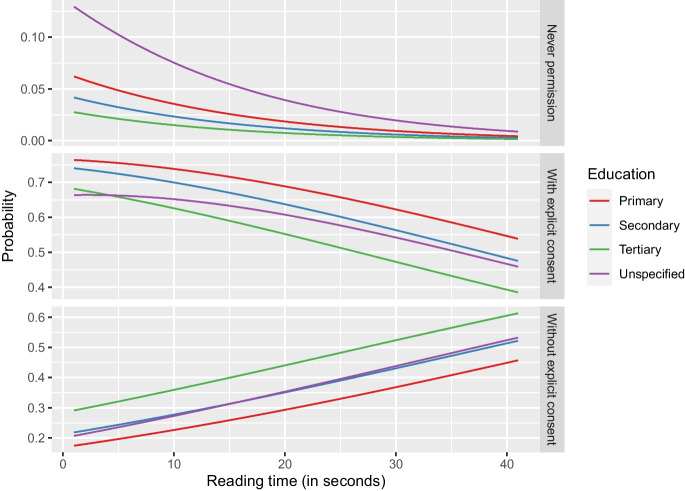
Should patients approve use of retrospective data by a hospital? Specified for level of education and reading time of an explanation of retrospective radiology

**Fig. 3:**
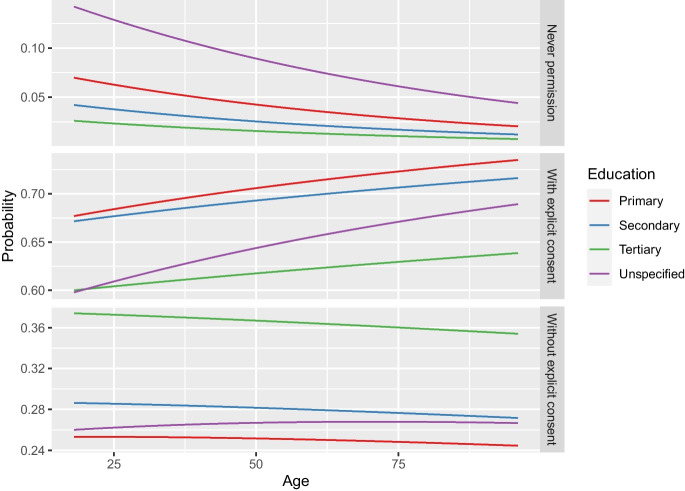
Should patients approve use of retrospective data by a hospital? Specified for level of education and age

**Fig. 4:**
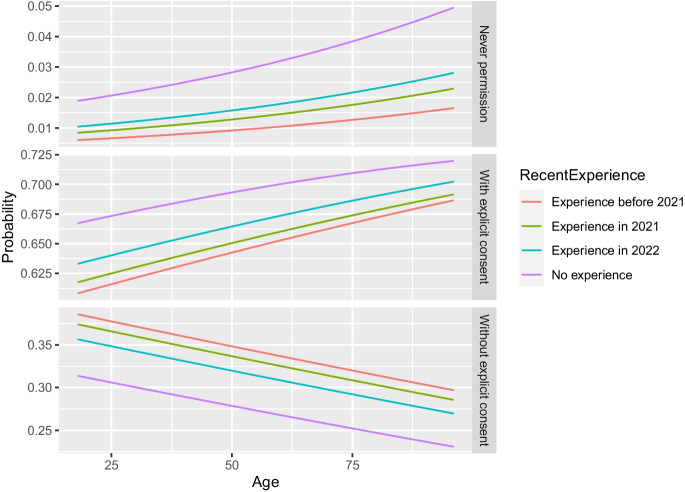
Should patients approve use of retrospective data by a hospital? Specified for level of experience with radiological examinations and age

Figures 1, 2, 3 and 4 further illustrate these outcomes for consent with data in the hospital: especially respondents with reading times below twenty seconds were more likely to choose “never permission” or “permission with explicit patient consent” (Figs. 1, and 2). In addition, male respondents were more likely to select “permission without explicit patient consent” than females, whereas females were more likely to choose “never permission” and “permission with explicit patient consent” than males (Fig. 1). Increase in reading time showed increased selection of “permission without explicit patient consent” for all levels of education (Fig. 2). Differences in odds of choosing “never permission” were mostly found for respondents with an unspecified level of education (Figs. 2 and 3) and for respondent with no experience in radiological examination, for whom the odds of choosing “never permission” appeared to increase with age (Fig. 4). Selection of “permission without explicit patient consent” seemed to slightly decrease with increasing age, but there was a contrast between tertiary versus other levels of education (Fig. 3). The supplemental materials (Figs. 1a–2e) include figures illustrating outcomes for all five institutions.

## Discussion

The results of this study show that the view of the general population on the retrospective use of diagnostic imaging data for research purposes is variable, depending on the setting. Although the vast majority would allow hospitals to perform retrospective research with diagnostic imaging data (97.4 per cent), this proportion drops somewhat for universities (89.7 per cent), other non-commercial institutions (82.4 per cent), and government agencies (79.5 per cent), while only a minority would allow commercial firms to use their diagnostic imaging data (36.5 per cent). This finding may be explained by the fact that patients already have a relationship with their hospital based on some degree of trust and that this trust decreases in institutions with which such a relationship did not exist. It also shows that government agencies are not completely trusted, especially by respondents with primary education as highest level of education completed, and that faith in for-profit companies is generally lacking. Another finding of this study is that the majority of participants indicated their explicit consent to be required before any diagnostic imaging data can be used for retrospective research. Importantly, however, whether or not participants deemed consent necessary depended on two variables, namely the time spent on reading the explanation about retrospective radiology research in this survey and level of education. Participants who spent more time on reading the explanation about retrospective radiology research more frequently indicated no need for their approval to execute such studies. In fact, when including only those participants (32.0 per cent) who spent at least the minimum reading time to be able to comprehend the text (12.3 s), the preference for a waiver of consent increased by ten percentage points, to 41.0 per cent. This is also in line with the finding that a majority of patients do not read informed consent texts (Özhan et al. 2014). The variance in time spent on reading the explanation about retrospective radiology research can be explained by the fact that the participants previously answered several other questions related to radiology in this survey (that were not part of the present study) and that some participants may have been impatient or considered it unnecessary to read the full explanation. Younger respondents and respondents with a lower level of education were less likely to take (sufficient) time for reading the text than older and higher educated respondents. People who had completed a higher level of education also more frequently indicated informed consent to be unnecessary compared to lower educated people. This is in line with a large body of previous literature that has suggested that education leads to higher trust (cited in Wu 2021), where the Netherlands is in the top ten countries with a positive correlation, ranking close to smaller countries like Slovenia and Trinidad and Tobago, but also larger countries like the United States. Finally, it should be noted that participants’ view on the retrospective use of diagnostic radiology data was similar to their view on the retrospective use of interventional radiology data.

Our findings may have two potential practical implications. First, policymakers may perhaps be more lenient towards permitting the use of deidentified radiology data without requiring patient informed consent, provided the situation concerns a hospital setting and patients are well informed about what retrospective radiology research entails (in this situation, a substantial proportion of respondents indicated explicit patient consent to be unnecessary). This approach is not in line with the GDPR article 5, where it is stated that data that is “collected for specified, explicit and legitimate purposes and not further processed in a manner that is incompatible with those purposes” (European Commission 2022). However, studies have shown that, under certain conditions, people are nevertheless willing to share data for health research (Kalkman et al. 2022), and they are also less likely to revoke once having given consent (Kreuter et al. 2020). Therefore, patients may be assumed to have no objections to having their imaging data being retrospectively investigated unless indicated by an opt-out option that should be readily available to patients and visible to researchers. However, the passive nature of opt-out causes an opt-in procedure a preferred standard (Lutomski and Manders 2024). Moreover, opt-out procedures may increase administrative burden (Marcotte et al. 2023). In addition, for patients to be able to make a well-informed decision to make use of this opt-out option, hospitals or radiology departments may be encouraged to enclose additional information on this topic in patient brochures and/or pamphlets, and by verbal communication to those who undergo medical imaging. This information should be adapted to those with lower levels of education, as our research has shown lower educated people to be generally more reluctant towards letting researchers retrospectively investigate their imaging data. Especially interactive components in the informed consent process to facilitate understanding are important (Glaser et al. 2020). Second, for all other institutions that do not concern a hospital (i.e. universities not linked to a hospital, other non-commercial institutions, government agencies, and particularly commercial firms), the support for an informed consent waiver for retrospective radiology research drops dramatically, and for these institutions, it can be argued that an “opt-out option” would be inappropriate, and that all patients should be actively approached and asked for informed consent to use their imaging data.

This present study had some limitations. First, our study was performed among participants who can be considered representative of the Dutch population, and it remains unknown if our findings are also applicable to other countries. Second, our study did not investigate the general population’s view on more complex issues such as situations in which non-commercial investigators work together with commercial partners and when researchers aim to investigate imaging data linked to genetic data. Third, for the sake of not introducing too much complexity, we did not specifically inform participants about the difference between fully anonymized and pseudonymized data. Fourth, we speculated about potential practical implications of our findings (i.e., assumption of no patient objection for retrospective radiology research by hospitals unless indicated by patients and assumption of patient objection for retrospective radiology research by all other institutions unless explicitly permitted by patients), but whether or not the population would actually accept these scenarios remains unclear. Furthermore, it should also be emphasized that previous research on this topic has been completely lacking so far. Therefore, the results of our study should be considered as a baseline measurement of the general population’s view on retrospective radiology research and to serve as a basis for further studies.

In conclusion, the general population permits retrospective radiology research by hospitals, and a substantial proportion indicates explicit patient consent to be unnecessary when understanding what retrospective radiology research entails. However, the general population’s support for the unrestricted retrospective use of imaging data for research purposes without patient consent decreases for universities not linked to hospitals, other non-commercial institutions, government agencies, and particularly commercial firms.

## Supplementary Information

Below is the link to the electronic supplementary material.

Supplementary file1 (DOCX 165 KB)

## Data Availability

All data is available online on the website of the LISS Panel, under project 322 via this link: https://www.dataarchive.lissdata.nl/study-units/view/1403.

## Declarations

This research is part of a project funded by the Open Data Infrastructure for Social Science and Economic Innovations (ODISSEI) in the Netherlands.

Ethical approval for the procedures in the LISS Panel was given by the board of overseers (LISS Board of Overseers 2022).
