# Supplementary material for: Retrospective Radiology Research: Do We Need Informed Patient Consent?
Source: J Bioeth Inq. 2024 Aug 19;22(1):175–85. doi: 10.1007/s11673-024-10368-6 (PMC12222269; doi:10.1007/s11673-024-10368-6)
Supplement: Supplementary file 1 — Supplementary file1 (DOCX 165 KB) [file 11673_2024_10368_MOESM1_ESM.docx]

Supplemental materials

**Table 2a Descriptive statistics of consent with retrospective use of interventional radiology data**

|  | All respondents  (n=2409) | | | Respondents with reading time > 12.3 seconds (n=769) | | |
| --- | --- | --- | --- | --- | --- | --- |
|  | Never permission | Permission *with* explicit patient consent | Permission *without* explicit patient consent | Never permission | Permission *with* explicit patient consent | Permission *without* explicit patient consent |
| By the hospital | 73 (3.0%) | 1574 (65.4%) | 760 (31.6%) | 13 (1.7%) | 438 (56.9%) | 318 (41.3%) |
| By a university | 263 (10.9%) | 1745 (72.5%) | 399 (16.6%) | 59 (7.7%) | 510 (66.2%) | 200 (26.0%) |
| By other non-commercial institutions | 451 (18.7%) | 1629 (67.7%) | 327 (13.6%) | 114 (14.8%) | 488 (63.4%) | 167 (21.7%) |
| By commercial firms | 1521 (63.2%) | 829 (34.4%) | 57 (2.4%) | 509 (66.1%) | 235 (30.5%) | 25 (3.2%) |
| By government agencies | 506 (21.0%) | 1541 (64.0%) | 360 (15.0%) | 152 (19.7%) | 464 (60.3%) | 153 (19.9%) |

Figures 1a-2e

*Figure 2a Should patients approve use of retrospective data by a hospital?*

*Specified for level of education and reading time of an explanation of retrospective radiology research*

*Figure 1a Should patients approve use of retrospective data by a hospital?*

*Specified for gender and reading time of an explanation of retrospective radiology research*

*Figure 2b Should patients approve use of retrospective data by a university?*

*Specified for level of education and reading time of an explanation of retrospective radiology research*

*Figure 1b Should patients approve use of retrospective data by a university?*

*Specified for gender and reading time of an explanation of retrospective radiology research*

*Figure 1c Should patients approve use of retrospective data by a non-commercial organization?*

*Specified for gender and reading time of an explanation of retrospective radiology*

*Figure 2c Should patients approve use of retrospective data by a non-commercial organization?*

*Specified for level of education and reading time of an explanation of retrospective radiology*

*Figure 2d Should patients approve use of retrospective data by a commercial organization?*

*Specified for level of education and reading time of an explanation of retrospective radiology*

*Figure 1d Should patients approve use of retrospective data by a commercial organization?*

*Specified for gender and reading time of an explanation of retrospective radiology*

*Figure 2e Should patients approve use of retrospective data by a governmental organization?*

*Specified for level of education and reading time of an explanation of retrospective radiology*

*Figure 1e Should patients approve use of retrospective data by a governmental organization?*

*Specified for gender and reading time of an explanation of retrospective radiology*
